# GENETICS AND MECHANISMS OF HUMAN AGING AND FRAILTY

**DOI:** 10.1093/geroni/igz038.809

**Published:** 2019-11-08

**Authors:** David Melzer, Luigi Ferrucci

**Affiliations:** 1 University of Exeter, Exeter, Devon, United Kingdom; 2 Translational Gerontology Branch, Intramural Research Program, National Institute on Aging, National Institutes of Health, Baltimore, Maryland, United States

## Abstract

Great progress has been made recently in identifying the genetic and likely biological mechanisms of aging traits in humans, thanks to the very large UK Biobank cohort of 500,000 persons. This symposium will discuss new results for known genetic loci influencing aging, including in-depth analysis of the APOE e2 “protective” allele and aging outcomes, and will highlight new pathways for follow-up. Identifying genetic variants associated with frailty (accumulation of deficits) is implicating specific pathways with possible causal effects on premature frailty, with particular emphasis on neurological traits. Large-scale analysis of physical frailty – here parametrized as muscle weakness – is shedding light on specific mechanisms that are divergent from those associated with the Rockwood-like analysis, with evidence for sex-specific pathways. At the end of this symposium, the audience should be able to better understand the processes that potentially drive aging and frailty in older adults, and the possibilities for future diagnostic and treatment modalities for delaying or reversing aging and frailty in older adults.

